# Single-cell motility and gene expression signature as predictors of the overall survival of act in melanoma patients

**DOI:** 10.1186/2051-1426-3-S2-P33

**Published:** 2015-11-04

**Authors:** Melisa Martinez-Paniagua, Cara Haymaker, Jay R Adolacion, Laszlo Radvanyi, Patrick Hwu, Badrinath Roysam, Chantale Bernatchez, Navin Varadarajan

**Affiliations:** 1 University of Houston, Houston, TX, USA; 2 UT MD Anderson Cancer Center, Houston, TX, USA; 3 University of Houston, University of the Philippines, Houston, TX, USA; 4 Moffit Cancer Center, Tampa, FL, USA

## Background

Adoptive cell therapy (ACT) of tumor infiltrating lymphocytes (TIL) has shown the ability to induce complete regression of stage IV melanoma. Two challenges associated TIL ACT are that (i) T cells are capable of different anti-tumor effector functions including cytotoxicity, cytokine secretion, and homing to target tissues, and currently no methodologies exist that can inform on all of these functions for a given (single) cell, and (ii) the relative contributions of each of these effector functions to the overall anti-tumor effect remains un-quantified.

## Methods

Here, we evaluated a set of four infusion products from patients treated with TIL ACT: two Complete Responders (CR) and two patients with Progressive Disease (PD) with high percentages of CD8^+^ T cell population and with established autologous primary tumor cell lines.

## Results

Flow cytometry analysis of the tumor reactive TIL showed no difference in the frequency of TIL secreting IFN-γ or degranulating in response to the autologous tumor. Utilizing Timelapse Imaging Microscopy In Nanowell Grids (TIMING), we demonstrate that while the frequencies of individual TIL participating in killing of autologous tumor cells is equivalent across the donors, TIL from CR patients demonstrated a longer duration of conjugation prior to killing the tumor cell (CR 211 ± 7 min vs PD 131 ± 5 min). Furthermore, both in the presence and absence of tumors cells, the motility of the TIL was significantly higher (CR 4.7 ± 0.2 µm vs PD 2.9 ± 0.1 µm). Multiplexed single-cell gene-expression profiling of the TIL, classified based on activated functional states (CD69/CD107a/IFN-γ) demonstrated that the functional classification was not useful in clustering cells from different donors but rather, clustering of cells was dictated by the donor regardless of function. Lastly, we demonstrate that a core signature of the activated TIL consisting of *CD37, CD44, IL7R, CD28, CD27, IFNGR1, TIM-3, MAPK1* and *MTOR* were up-regulated in CR patients and the levels of *TGFB1, BCL2, IL2RG, PDRM1* and *IL12RB2* were up-regulated in PD patients.

## Conclusions

In summary, our results demonstrate that single cell profiling may be useful in determining not only the transcriptional signatures of TIL with favorable clinical responses but also uncover simple physical attributes like basal motility that might serve as a surrogate marker for activated/functional TIL.

